# Enhanced Glycolysis Is Required for Antileishmanial Functions of Neutrophils Upon Infection With *Leishmania donovani*

**DOI:** 10.3389/fimmu.2021.632512

**Published:** 2021-03-19

**Authors:** Mareike Ohms, Carolina Ferreira, Hauke Busch, Inken Wohlers, Ana Cristina Guerra de Souza, Ricardo Silvestre, Tamás Laskay

**Affiliations:** ^1^Department of Infectious Diseases and Microbiology, University of Lübeck, Lübeck, Germany; ^2^Life and Health Sciences Research Institute (ICVS), School of Medicine, University of Minho, Braga, Portugal; ^3^ICVS/3B's-PT Government Associate Laboratory, Guimarães, Portugal; ^4^Medical Systems Biology Division, Lübeck Institute of Experimental Dermatology and Institute for Cardiogenetics, University of Lübeck, Lübeck, Germany; ^5^Department of Cell Technology, Fraunhofer Research Institution for Marine Biotechnology and Cell Technology (EMB), Lübeck, Germany

**Keywords:** neutrophils, glucose metabolism, glycolysis (glycolytic pathway), oxidative phosphorylation (OXPHOS), leishmania, host-directed therapy (HDT), 2-deoxyglucose, transcriptome (RNA-seq)

## Abstract

Visceral leishmaniasis (VL) is a fatal parasitic disease if untreated. Treatment options of VL diminish due to emerging drug resistance. Although the principal host cells for the multiplication of *Leishmania* are macrophages, neutrophils are the first cells infected with the parasites rapidly after parasite inoculation. *Leishmania* can survive in neutrophils despite the potent antimicrobial effector functions of neutrophils that can eliminate the parasites. Recently, the growing field of immunometabolism provided strong evidence for the therapeutic potential in targeting metabolic processes as a means of controlling immune effector functions. Therefore, the understanding of the immunometabolic profile of neutrophils during *Leishmania* infection could provide new promising targets for host-directed therapies against VL. To our knowledge, this is the first study addressing the bioenergetics profile of *L. donovani*-infected primary human neutrophils. Transcriptome analysis of *L. donovani*-infected neutrophils revealed an early significant upregulation of several glycolytic enzymes. Extracellular flux analysis showed that glycolysis and glycolytic capacity were upregulated in *L. donovani*-infected neutrophils at 6 h post infection. An increased glucose uptake and accumulation of glycolytic end products were further signs for an elevated glycolytic metabolism in *L. donovani*-infected neutrophils. At the same time point, oxidative phosphorylation provided NADPH for the oxidative burst but did not contribute to ATP production. Inhibition of glycolysis with 2-DG significantly reduced the survival of *L. donovani* promastigotes in neutrophils and in culture. However, this reduction was due to a direct antileishmanial effect of 2-DG and not a consequence of enhanced antileishmanial activity of neutrophils. To further address the impact of glucose metabolism during the first days of infection *in vivo*, we treated C57BL/6 mice with 2-DG prior to infection with *L. donovani* and assessed the parasite load one day and seven days post infection. Our results show, that seven days post-infection the parasite load of 2-DG treated animals was significantly higher than in mock treated animals. This data indicates that glycolysis serves as major energy source for antimicrobial effector functions against *L. donovani*. Inhibition of glycolysis abrogates important neutrophil effector functions that are necessary the initial control of *Leishmania* infection.

## Introduction

Leishmaniasis is a vector-borne neglected tropical disease transmitted by sand flies and caused by obligate intracellular protozoa of the genus *Leishmania* (1). Visceral leishmaniasis (VL), also known as Kala-Azar, is the most fatal form of leishmaniasis and has an incidence of estimated 400,000 cases per year (source: Centers for Disease Control and Prevention). There are several treatment options of leishmaniasis of which pentavalent antimonials has been considered the gold standard for decades (2). However, massive side effects and an increase in drug resistance mark the urgent need for new therapeutic strategies and a better understanding of host-pathogen interactions (3). Neutrophils are the most abundant white blood cells and provide the first line of defense against invading pathogens. Equipped with an arsenal of effector functions, they rapidly migrate to sites of infection to eliminate pathogens. Prominent effector functions include phagocytosis, oxidative burst, degranulation and the release of neutrophil extracellular traps (NETs) (4).

Several studies pointed out the crucial role of neutrophils in leishmaniasis outcome. Neutrophils are massively and rapidly recruited to the site of infection and are the first cells to encounter the parasites (5, 6). They participate in their elimination through the production of reactive oxygen species (ROS), the release of azurophilic granules that contain antimicrobial proteins such as neutrophil elastase (NE) and myeloperoxidase (MPO) (7, 8). In addition, neutrophils can release NETs (9), which can kill some *Leishmania* spp. (10). In contrast to their well-described protective role in many infections, neutrophils can play a detrimental role in the manifestation of leishmaniasis (11, 12). Although macrophages serve as definitive host cell for *Leishmania*, neutrophils can provide a transient shelter for the intracellular pathogen (13). Parasitized neutrophils can function as a “Trojan horse” (14) or “Trojan rabbit” strategy (15) to transfer *Leishmania* to macrophages.

Metabolic reprogramming of immune cells is well described in infectious diseases (16–18) and competition between the host and the pathogen for nutritional substrates exists due to their similar nutritional requirements (18). It is well-established that *Leishmania* infection has a profound effect on host cell metabolism and this is intimately correlated with intracellular parasite survival (19, 20). In particular, we have previously shown the switch from early glycolysis to late oxidative phosphorylation under the control of the SIRT1-AMPK axis in *L. infantum*-infected murine macrophages (19). Inhibition of the SIRT1-AMPK axis was effective in reducing the parasitic load both *in vitro* and *in vivo* (19). Herein, we demonstrate that *L. donovani*-infected neutrophils display an upregulation of genes involved in glycolytic metabolism along with an increase in glucose consumption and glycolytic rates. *In vivo* blocking of glycolysis by using the glucose analog 2-DG resulted in increased susceptibility to infection associated with increased parasite burdens in the liver and the spleen of 2-DG-treated animals seven days post-infection. Taken together, our results demonstrate that glycolytic metabolism is essential for the initial control of *L. donovani* infection.

## Materials and Methods

### Ethics Statement

Blood collection was conducted with the agreement and written consent form of each participant and was approved by the Ethical Committee of the University of Lübeck (18-186). The *in vivo* experiments using animal models were conducted with the approval of the UMinho Ethical Committee (process no. SECVS 074/2016) and complied with the guidelines of the Committee and National Council of Ethics for the Life Sciences (CNECV). CF and RS have an accreditation for animal research given from Portuguese Veterinary Direction (Ministerial Directive 1005/92).

### Isolation of Primary Human Neutrophils

Peripheral blood was collected by venipuncture from healthy adult volunteers using lithium–heparin collection tubes (S-Monovette® 9 ml LH, Sarstedt, Nümbrecht, Germany). Blood was layered on a two-layer density gradient consisting of an upper layer of Histopaque® 1077 (Sigma Aldrich, Steinheim, Germany) and a lower layer of Histopaque® 1119 (Sigma Aldrich) and centrifuged for 5 min at 300 × g followed by 25 min at 800 × g. Cells from the upper layer consisting mainly of lymphocytes and monocytes were discarded. The granulocyte-rich lower layer was collected, leaving the erythrocyte pellet at the bottom of the tube. Granulocytes were washed once in 1 × Dulbecco's phosphate-buffered saline (DPBS) (Thermo Fisher, Germany) for 10 min at 800 × g, resuspended in complete medium [RPMI 1640 Medium (Sigma Aldrich) supplemented with 2 mM L-glutamine (Merck, Darmstadt, Germany), 10 mM 4-(2-hydroxyethyl)-1-piperazineethanesulfonic acid (HEPES; Life Technologies, Darmstadt, Germany), 10% heat-inactivated (FCS; Gibco, Germany), 100 U/ml penicillin, and 100 μg/ml streptomycin (Biochrom, Berlin, Germany)] and further fractionated on a discontinuous Percoll® (GE Healthcare, Braunschweig, Germany) gradient consisting of layers with densities of 1.105 g/ml (85 %), 1.100 g/ml (80 %), 1.087 g/ml (70 %), and 1.081 g/ml (65 %). After centrifugation for 25 min at 800 × g, the interface between the 80 and 70% Percoll® layers was collected. The cells were washed once in 1× DPBS for 10 min at 800 × g and resuspended in complete medium to a concentration of 5 × 10^6^ cells/ml. All described procedures were conducted at room temperature and under sterile conditions. Cell counting was conducted with a hemocytometer (Imp. Neubauer, 0.0025 mm^2^, depth 0.100 mm, VWR, Dresden, Germany) and crystal violet staining. The preparations contained ≥99% granulocytes, of which >95% were neutrophils and 1–4 % were eosinophils, as determined by Giemsa staining (Diff Quick Fix, Medion Diagnostics, Berlin, Germany) of cytocentrifuged (Shandon) samples.

### *Leishmania donovani* Culture

Virulent *L. donovani* promastigotes (strain MHOM/IN/82/Patna 1) were cultivated in Schneider's Drosophila Medium with L-glutamine (Genaxxon, Ulm, Germany) supplemented with 10% FCS, 100 U/ml penicillin, 100 μg/ml streptomycin, and 2 % sterile filtered human urine at 27°C in a humidified air atmosphere containing 5% CO_2_. For seeding of cultures, a parasite density of 1 × 10^6^ cells/ml was used. The parasite counting was conducted in a hemocytometer with a chamber depth of 0.02 mm (0.0025 mm^2^, depth 0.02 mm, VWR). The culture was considered to be in the stationary phase 72 h after seeding. Serial passaging was conducted until passage 10.

### *In vitro* Infection of Neutrophils With *Leishmania donovani* Promastigotes

The parasites were washed once with PBS to remove remaining Leishmania culture medium. Neutrophils and *L. donovani* promastigotes were coincubated in complete medium at a parasite-neutrophil ratio of 10:1 and a neutrophil density of 5 × 10^6^ cells/ml for 3 h at 37°C in a humidified air atmosphere containing 5 % CO_2_. Subsequently, the infection rate was assessed on Giemsa-stained cytocentrifuged samples. The cells were washed six times in 1 × PBS at 400 × g for 10 min to remove extracellular *Leishmania*. Infected cells were again adjusted to 5 × 10^6^ cells/ml. The assays were set up with the very same cells from the individual blood donors. A part of the freshly isolated neutrophils was infected with *Leishmania donovani*, the other part of the cells remained uninfected. The only difference between the infected and uninfected population is the exposure to and the intracellular presence of *Leishmania* parasites in the infected sample. The assays (*n* = 3-8) were carried out with cells isolated from different blood donors.

### 2-NBDG Uptake

To analyze the uptake of glucose, the fluorescent glucose analog 2(N-(7-nitrobenzen-2oxa-1,3-diazol-4-yl)amino)-2-deoxyglucose (2-NBDG) (Cayman, Michigan, USA) was used. Neutrophils were adjusted to 5 x 10^6^ cells/ml in glucose-free XF-assay medium (SeahorseBiosciences, Denmark) supplemented with 100 μg/ml 2-NBDG. The cells were incubated for 10 min at 37°C and 5% CO_2_. Subsequently, the cells were washed twice and resuspended in FACS buffer [1 x DPBS (Thermo Fisher Scientific, Waltham, Massachusetts, USA) + 1 % human serum + 1 % BSA (Sigma-Aldrich, Steinheim, Germany) + 0.01 % sodium azide (Merck, Darmstadt, Germany), sterile filtered, pH 7.2]. 2-NBDG uptake was assessed by flow cytometry.

### Lactate Assay

The metabolite L(+)-lactate was detected by the lactate assay kit (Sigma-Aldrich, Steinheim, Germany) in cell-free culture supernatants. The supernatants of corresponding samples were filtered through an Amicon® 10 kDa molecular weight cutoff ultracentrifuge filter (Merck, Darmstadt, Germany) for 15 min at 21 000 x g at RT to remove the lactate dehydrogenase. The lactate assay was performed with 5 μl of the filtrate in accordance with the manufacturer's instructions. The lactate concentration was calculated by interpolation of a standard curve and after background correction by subtraction of medium only values.

### Pyruvate Assay

The metabolite pyruvate was detected by the pyruvate assay kit (Sigma-Aldrich, Steinheim, Germany) in whole cell lysates. 1 × 10^6^ neutrophils were resuspended in 500 μl Aqua dest. and boiled for 5 min at 100°C. The pyruvate assay was performed with 5 μl of the lysate in accordance with the manufacturer's instructions. The pyruvate concentration was calculated by interpolation of a standard curve.

### Detection of Reactive Oxygen Species (ROS)

The sum of intra- and extracellular MPO-derived ROS was measured by using the luminol-amplified chemiluminescence assay. Neutrophils (400,000 cells/sample) were resuspended in complete medium without FCS and seeded in a flat-bottom white 96-well plate (NuncTM F96 MicroWellTM polystyrol plate, Thermo Fisher). Subsequently, 60 μM luminol (Invitrogen, Germany) was added, and the cells were stimulated by the addition of 20 nM phorbol 12-myristate 13-acetate (PMA; Sigma Aldrich). The chemiluminescence resulting from ROS release was analyzed immediately by an Infinite M200pro-Tecan reader (Tecan, Männedorf, Switzerland) and Tecan i-control 1.7 software. ROS release was monitored every minute for a period of 1 h at 37°C and 5% CO_2_.

### Bioenergetic Profile of *L. donovani*-Infected Neutrophils

On the day before the experiment, 1 ml of XF calibrant solution (Agilent, California, USA) was added to each well of a Seahorse XF24 sensor cartridge (Agilent, California, USA). The filled Seahorse XF24 sensor cartridge was incubated at 37°C without CO_2_ overnight. Additionally, a Seahorse XF24 cell culture microplate (Agilent, California, USA) was coated. For coating 1.4 ml of 0.1 M sodium bicarbonate buffer and 17 μl Corning™ Cell-Tak (Corning Life Sciences, Amsterdam, The Netherlands) were mixed and 50 μl were pipetted to the bottom of each well. After incubating the plate 20 min at RT, the wells were washed twice with 500 μl Aqua dest. and before usage the plate was air-dried under the laminar flow workbench. For Seahorse XF glycolysis stress test XF assay medium (Agilent, California, USA) was preheated at 37°C in a water bath and supplemented with 2 mM L-glutamine. The pH was adjusted to 7.35. For Seahorse XF Mito stress test 37°C-preheated XF assay medium was supplemented with 1 mM sodium pyruvate (PAN-Biotech, Aidenbach, Germany), 2 mM L-glutamine and 5 mM glucose (Thermo Fisher Scientific, Waltham, Massachusetts, USA). The pH was adjusted to 7.4. Neutrophils were adjusted to 25 x 10^6^ cells/ml in the corresponding supplemented XF assay medium and 100 μl of cells were added to each well except the four blank controls, which were filled with XF assay medium. For adherence of cells the plate was centrifuged at 40 x g without brake until the desired speed was reached. Afterwards the plate was turned 180° clockwise and was centrifuged at 80 x g. Finally, 425 μl of the corresponding supplemented XF assay medium were added to each well. The plate was incubated 60 min at 37°C without CO_2_ and inserted into the Seahorse XF24 Analyzer. The real time measurement of bioenergetic profile was obtained under basal conditions and in response to glucose (5 mM), oligomycin (1 μM) (Sigma-Aldrich, Steinheim, Germany), 2-desoxyribose (2-DG) (10 mM) (Sigma-Aldrich, Steinheim, Germany), fluoro-carbonyl cyanide phenylhydrazone (FCCP) (1μM) (Cayman Chemical, Ann Arbor, Michigan, USA), Rotenone (1 μM) (Cayman Chemical, Ann Arbor, Michigan, USA) and Antimycin A (1 μM) (Sigma-Aldrich, Steinheim, Germany). For Seahorse XF glycolysis stress test the acidification of the medium was measured directly by the Seahorse XF24 Analyzer and reported as the extracellular acidification rate (ECAR).

### Quantification of *Leishmania donovani* Survival *in vitro*

A limiting dilution culture assay was used to detect viable *L. donovani* parasites in neutrophils and survival of *L. donovani* parasites as described previously (21). Briefly, serial 1.5-fold dilutions of *L. donovani*-infected neutrophil suspensions (5 × 10^6^ cells/ml) or *L. donovani* promastigotes (25 x 10^6^ cells /ml) were plated in four replicates in 96-well flat-bottom microtiter plates containing Schneider's Drosophila Medium. The plates were incubated at 27°C in humidified air atmosphere containing 5% CO_2_ for 10–14 days. The growth of *L. donovani* promastigotes was detected microscopically. The last dilution resulting in a growth of parasites in > 50 % of the wells is given as a quantitative measure of the parasite load in the neutrophil cell suspension.

### Mice and Experimental *L. donovani* Infection

C57BL/6 mice were purchased from Charles River Laboratories and maintained in accredited animal facilities at the Life and Health Sciences Research Institute (ICVS). Males with 8–12 weeks were used for *in vivo* experiments. Mice were infected with 1.0 x 10^8^ (intraperitoneal route) stationary *L. donovani* promastigotes and weight and well-being was monitored during the infection. Intraperitoneal inoculation was used given that it results in a higher homogeneity of infections between animals. For glycolysis inhibition experiment, 2-DG was administered via intraperitoneal route, at days −3, −2, and −1 before infection (100 mg/kg). Control animals were equally injected with vehicle (sterile PBS). Experimental groups were kept in separated cages. Mice were euthanized at day 1 and day 7 post-infection and the liver and spleen were recovered for further analysis.

### Quantification of *Leishmania donovani* Survival *in vivo*

The liver and spleen were mechanically disrupted, and cells were recovered for DNA extraction. DNA was extracted using the phenol-chloroform-isoamyl alcohol method. Briefly, an aqueous suspension with approximately 1-3 x 10^6^ cells was mixed with a mixture of phenol-chloroform-isoamyl alcohol (25:24:1). After centrifugation, the aqueous phase was recovered and incubated overnight with 3M sodium acetate and absolute ethanol. The DNA pellet was washed twice with 70% ethanol and resuspended in RNase/DNase-free water. Parasite burden was assessed, using a TaqMan-based qPCR assay for detection and quantification of *L. donovani* kinetoplastid DNA as described previously (22).

### Flow Cytometry

The spleens were minced and forced through a 70-μm cell strainer (Corning Inc.) and red blood cells were lysed with ACK lysis buffer (0.15 MNH_4_Cl, 10 mM KHCO_3_, and 0.1 mM EDTA). The anti-mouse monoclonal antibodies used to perform this study were purchased from BioLegend (CA, USA) and surface staining was performed with the following antibodies: PE/Cy7 anti-mouse CD11b (clone M1/70APC), BV711 anti-mouse Ly6G (clone 1A8), PerCP/Cy5.5 anti-mouse Ly6C (clone HK14), APC anti-mouse I-A/I-E (clone M5/114.15.2) and BV605 anti-mouse CD62L (clone MEL-14). Samples were acquired on a LSRII flow cytometer (BD Biosciences) and data analyzed using FlowJo software (FlowJo LLC). The gating strategy for monocytes and neutrophils is shown in Supplementary Figure 2.

### RNA Isolation and Quality Control

RNA isolation from neutrophils was performed as previously described (23). Shortly, 10 x 10^6^ neutrophils were harvested in 2 ml reaction tubes and centrifuged at 300 x g for 5 min at RT. Further RNA isolation was performed by usage of the RNeasy mini kit (Qiagen, Hilden, Germany) in accordance with the manufacturer's protocol. The washing with 700 μl RW1 buffer as indicated in step 6 of the RNeasy mini kit protocol was not performed, instead an on column DNase digestion as suggested in the optional step 9 was carried out. The RNA was eluted in 30 μl of RNase-free water and stored at −70°C. The purity of the isolated RNA was assessed by measurement of the A260/A280 and the A260/A230 ratio with a UV/VIS spectrophotometer. For assessment of RNA integrity the RNA integrity number (RIN) of the isolated RNA was determined. The isolated RNA was diluted to 1 ng/μl in RNase-free water and boiled 2 min at 70°C. The RNA was further prepared as described in the protocol of the Agilent RNA 6000 Pico kit (Agilent, California, USA) and run on a chip in the Agilent 2100 Bioanalyzer. A RIN value ≥ 6.5 was defined as acceptable for transcriptome analysis. RNA sequencing was performed by the genome sequencing company Novogene (Hong Kong).

### Transcriptome Analyses

Paired-end RNA-Seq data was generated for 12 samples originating from three healthy donors referred to as A, B, and C. Sampled conditions are three biological replicates of uninfected neutrophils at time point 0, three biological replicates of uninfected neutrophils at time point 6 h and six replicates of neutrophils 6 h after infection with *L. donovani*. These latter six replicates originate from neutrophils of individuals A, B, and C, with each individual's neutrophils infected twice. The number of sequencing reads generated lies between 30 and 53 million (median 43 million). Quality of raw sequencing data was confirmed using fastqc (version 0.11.9) [http://www.bioinformatics.bbsrc.ac.uk/projects/fastqc]. Read length is 150 bases and insert size 250 bases. Sequencing reads were mapped with kallisto (version 0.46.1) (24) against a combined reference constructed from 191887 human transcript sequences of 40479 genes (ftp://ftp.ensembl.org/pub/release101/fasta/homo_sapiens/cdna/Homo_sapiens.GRCh38.cdna.all.fa.gz) and 8409 *L. major* transcript sequences of 8409 genes (ftp://ftp.ensemblgenomes.org/pub/protists/release_48/fasta/leishmania_major/cdna/Leishmania_major.ASM272v2.cdna.all.fa.gz). Between 20 and 42 million reads (median 30 million) aligned to human or *L. major*. On average, 81% of reads of uninfected samples and 64% of reads of infected samples were mapped. We also mapped against a combined reference constructed from human and *L. donovani* transcriptome (ftp://ftp.ensemblgenomes.org/pub/protists/release_48/fasta/protists_euglenozoa1_collection/leishmania_donovani_gca_000227135/cdna/Leishmania_donovani_gca_000227135.ASM22713v2.cdna.all.fa.gz), but since the mapping rate was comparable and *L. donovani* is less annotated, we used *L. major* in the following. Transcript expression was quantified using sleuth (version 0.30.0) (25). *Leishmania* genes are not expressed in uninfected cells, but amount to about half of estimated counts and three quarter of transcripts per million (TPM) in infected samples. Since estimated counts for human genes in infected conditions are thus low, lowly expressed human transcripts cannot be quantified reliably and we compute differential expression only for transcripts with more than 10 estimated counts in any sample. Normalized TPM values computed by sleuth are used for heatmap visualization. Significant differentially expressed transcripts between the six uninfected samples and the six infected samples were computed using the likelihood ratio test in sleuth. Sleuth's Wald test has been used to estimate effect size.

### KEGG Glycolysis Pathway-Centered Gene Expression

Human and *L. major* genes mapping to the KEGG (26) glycolysis pathway (KEGG release 95.2) have been obtained using the KEGG REST API and their transcript expression and differential expression results were extracted. Human transcripts and their expression effect estimates from sleuth have been visualized on the glycolysis KEGG reference pathway using pathview (version 1.30.0) (27).

### Statistical Analysis

If not stated differently, the presented data were generated from a minimum of three independent experiments with neutrophils isolated from different blood donors. Statistical analysis was performed with the GraphPad Prism software 6 using the one-way ANOVA followed by Sidak's *t*-test for multiple comparisons or unpaired *t*-test followed by Welch's correction. A *p*-value ≤ 0.05 was considered statistically significant.

## Results

### Human Genes on the Main Glycolytic Axis Are Upregulated in Neutrophils 6 h After *L. donovani* Infection

**Figure 1:**
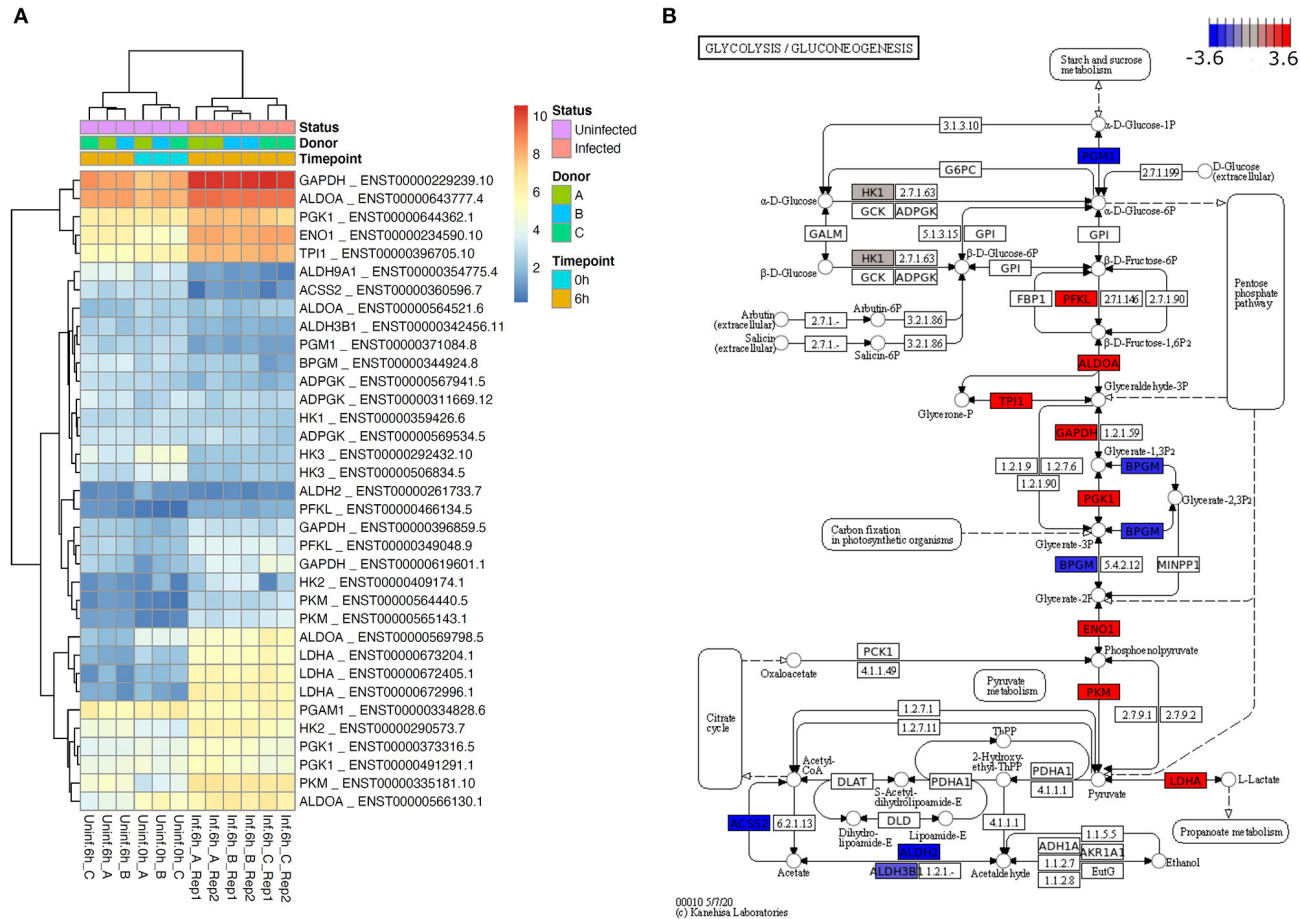
*L. donovani* induces enhanced glycolysis in neutrophils. **(A)** Heatmap of transcripts of selected glycolytic genes for six samples without and six samples 6 h after *L. donovani* infection. Displayed expression values have been normalized across samples by sleuth and are provided as log2 transcripts per million (log2 TPM). Shown are transcripts of human glycolytic genes according to KEGG pathway glycolysis if this transcript (i) is significantly differentially expressed according to sleuth likelihood ratio test with multiple testing corrected *p*-value, i.e., q-value, <0.05 and (ii) is highly expressed (sleuth mean of natural log counts ≥5). Heatmap annotation shows that samples cluster primarily by condition (infected vs. uninfected) and secondarily by donor (A, B, C). A heatmap of the highest expressed transcript of every gene that could be mapped to the KEGG glycolysis pathway (human as well as *L. major*) is provided in Supplementary Figure 3. **(B)** Estimated effect sizes according to sleuth Wald test for the most highly expressed, significant transcript of genes mapping to KEGG pathway glycolysis (colored are only genes with at least one transcript having mean of natural log count ≥5). Highly expressed and significantly differentially expressed human genes shown in **(A)** constitute the major glycolytic axis, and consistently show an increase of expression in infected neutrophils with the exception of BPGM, which has opposite effect by similar order of magnitude.

It has been known for many decades that neutrophils almost only rely on glycolysis to obtain energy (28). However, the role of glycolysis in neutrophils during infections has not been addressed so far. Our first aim of this study was therefore, to investigate the glycolytic host cell metabolism of *L. donovani*-infected neutrophils after 6 h of infection. For this approach, we used RNA-Seq analysis. Mapping of neutrophil transcripts to the KEGG glycolysis pathway revealed a substantial upregulation of genes involved in glycolysis as visualization by heatmap shows (Figure 1A). The commitment of neutrophils toward glycolysis was reflected by the upregulation of aldolase A (ALDOA), triosephosphate isomerase (TPI1), glyceraldehyde 3-phosphate dehydrogenase (GAPDH), phosphoglycerate kinase 1 (PGK1), enolase 1 (ENO1), and lactate dehydrogenase (LDH) (Figure 1B). Moreover, the transcription of two genes encoding the enzymes that control the rate of glycolysis, phosphofructokinase (PFKL) and pyruvate kinase (PKM), was significantly enhanced (Figure 1B). Taken together these data suggest that infection with *L. donovani* induces an enhanced glycolytic metabolism in neutrophils, which served as bioinformatics basis of our further *in vitro* and *in vivo* experiments.

### *L. donovani*-Infected Neutrophils Have an Increased Glycolytic Metabolism

**Figure 2:**
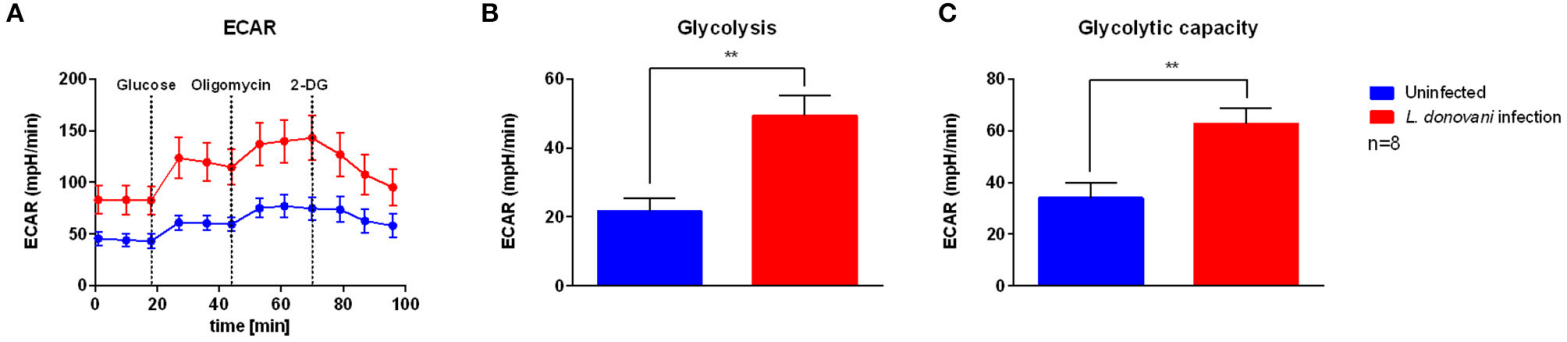
Glycolysis stress test profile of *L. donovani*-infected neutrophils. Primary human neutrophils were infected with *L. donovani* promastigotes (ratio 1:10) for 3 h at 37°C and 5% CO_2_. Uninfected cells served as control. The infection rate was determined by Giemsa staining of cytocentrifuged samples. Subsequently, free, non-ingested parasites were removed by washing. After 6 h post infection the glycolysis stress test profile was determined by using the Seahorse extracellular flux analyzer **(A)**. Extracellular acidification rate (ECAR) measurements following the sequential injection of 5 mM glucose, 1 μM oligomycin, and 10 mM 2-DG (dotted lines indicate injection time) were used to calculate key parameters of glycolytic function. Glycolysis **(B)** was calculated by subtraction of 2-DG-mediated ECAR from glucose-mediated ECAR. Glycolytic capacity **(C)** was calculated by subtraction of 2-DG-mediated ECAR from oligomycin-mediated ECAR. Bar graphs show mean ± SD (*n* = 8), ^**^p ≤ 0.01.

To address how *L. donovani* infection impacts neutrophil metabolism and bioenergetics state, the glycolytic metabolism was assessed by measuring the extracellular acidification rate (ECAR), a consequence of lactate production, by using live cell extracellular flux analysis. After 6 hpi, higher basal ECAR levels were observed in *L. donovani*-infected neutrophils compared to uninfected neutrophils (Figure 2A). As glycolysis is the main pathway used to supply ATP for the energy requirements of neutrophils (29–32), the key glycolytic parameters of *L. donovani*-infected neutrophils, namely glycolysis and glycolytic capacity, were calculated from ECAR values. Glycolysis and glycolytic capacity of *L. donovani*-infected neutrophils were increased after 6 hpi compared to uninfected neutrophils (Figures 2B,C). These data suggest that neutrophils rely on glycolysis during *L. donovani* infection.

### Infection With *L. donovani* Does Not Result in Enhanced ATP Production Due to Oxidative Phosphorylation

**Figure 3:**
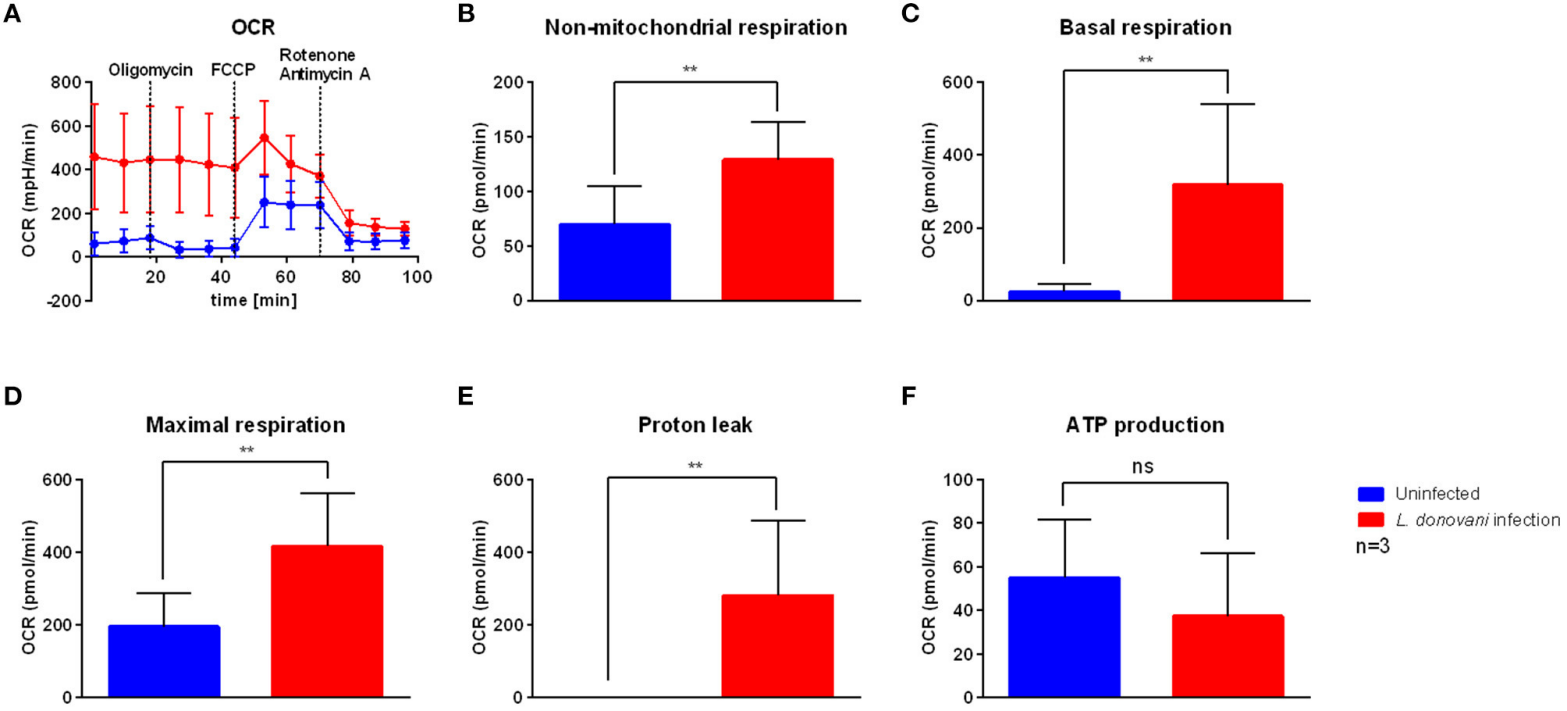
Mitochondrial stress test profile of *L. donovani*-infected neutrophils. Primary human neutrophils were infected with *L. donovani* promastigotes (ratio 1:10) for 3 h at 37°C and 5% CO_2_. Uninfected cells served as control. Successful infection was determined by Giemsa staining of cytocentrifuged samples. Subsequently, free, non-ingested parasites were removed by washing. After 6 h post infection the mitochondrial stress test profile was determined by using the Seahorse extracellular flux analyzer **(A)**. The measurement of basal oxygen consumption rate (OCR) was followed by sequential injections of 1 μM oligomycin, 1.5 μM FCCP, and 1 μM rotenone/antimycin A (dotted lines indicate injection time). OCR measurements were used to calculate key parameters of mitochondrial function. Non-mitochondrial respiration **(B)** was calculated as OCR after rotenone/antimycin A injection. Basal respiration **(C)** was calculated by subtraction of rotenone/antimycin A-mediated OCR from basal OCR. Maximal respiration **(D)** was calculated by subtraction of rotenone/antimycin A-mediated OCR from FCCP-mediated OCR. Proton leak **(E)** was calculated by subtraction of non-mitochondrial respiration from oligomycin-mediated OCR. ATP production **(F)** was calculated by subtraction of oligomycin-mediated OCR from basal OCR. Bar graphs show mean ± SD (*n* = 3), ^**^p ≤ 0.01, ns, not significant.

To examine the effect of *L. donovani* infection on host oxidative phosphorylation, an extracellular flux analysis of a mitochondrial stress test was used to determine key respiratory parameters, including non-mitochondrial respiration, basal respiration, maximal respiration, proton leak, ATP production, and spare respiratory capacity. The oxygen consumption rate (OCR) of *L. donovani*-infected neutrophils at 6 hpi was higher compared to uninfected neutrophils (Figure 3A). Additionally, non-mitochondrial respiration, basal respiration, maximal respiration and proton leak of *L. donovani*-infected neutrophils were significantly increased compared to uninfected neutrophils (Figures 3B–E). In contrast, no difference was observed on the mitochondrial ATP production of *L. donovani*-infected neutrophils when compared to uninfected cells (Figure 3F). These results indicated that *L. donovani*-infected neutrophils consume more oxygen that fuels the production of reactive oxygen species (ROS) to eliminate the intracellular pathogen.

### *L. donovani* Infection Results in Enhanced Glucose Uptake of Neutrophils

**Figure 4:**
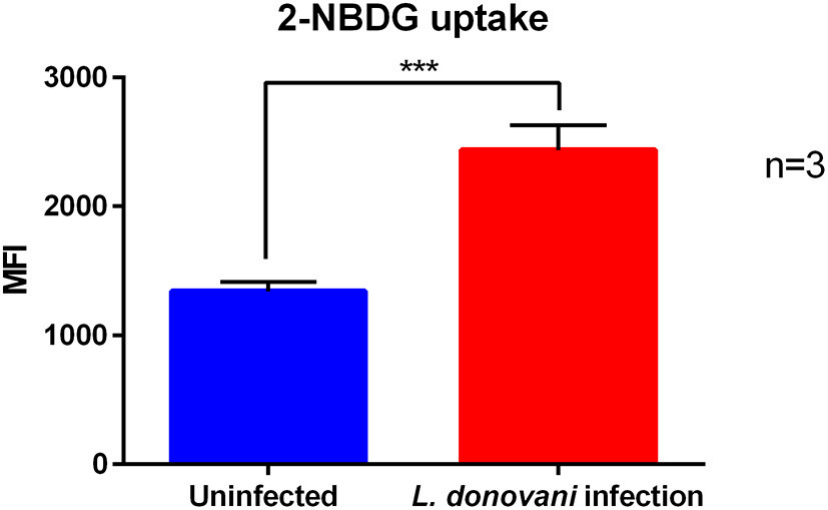
2-NBDG uptake of *L. donovani*-infected neutrophils. Primary human neutrophils were infected with *L. donovani* promastigotes (ratio 1:10) for 3 h at 37°C and 5 % CO_2_. Uninfected cells served as control. The infection rate was determined by Giemsa staining of cytocentrifuged samples. Subsequently, free, non-ingested parasites were removed by washing. After 6 h post infection the fluorescent glucose analog 2-NBDG was added to the cells in glucose-free medium for the last 10 min of incubation time and 2-NBDG uptake was analyzed by flow cytometry. The bar diagram shows the autofluorescence corrected mean fluorescence intensity (MFI) of 2-NBDG uptake ± SD (*n* = 3), ^***^p ≤ 0.001.

To fuel glycolysis glucose must be transported from the extracellular milieu into the cells. This task is accomplished either by sodium coupled glucose transporters (SGLTs) or by glucose facilitative transporters, the GLUT family (33). To complement our analysis, we measured the uptake of the fluorescent glucose analog 2-NBDG in *L. donovani*-infected and uninfected neutrophils by flow cytometry. After 6 hpi *L. donovani*-infected neutrophils displayed a significantly higher glucose uptake compared to uninfected neutrophils (Figure 4). The increased glucose uptake of neutrophils during *L. donovani* infection supports the view that neutrophils mainly rely on glycolysis during the infection with the intracellular parasite.

### *L. donovani*-Infected Neutrophils Exhibit Increased Levels of Lactate and Pyruvate

**Figure 5:**
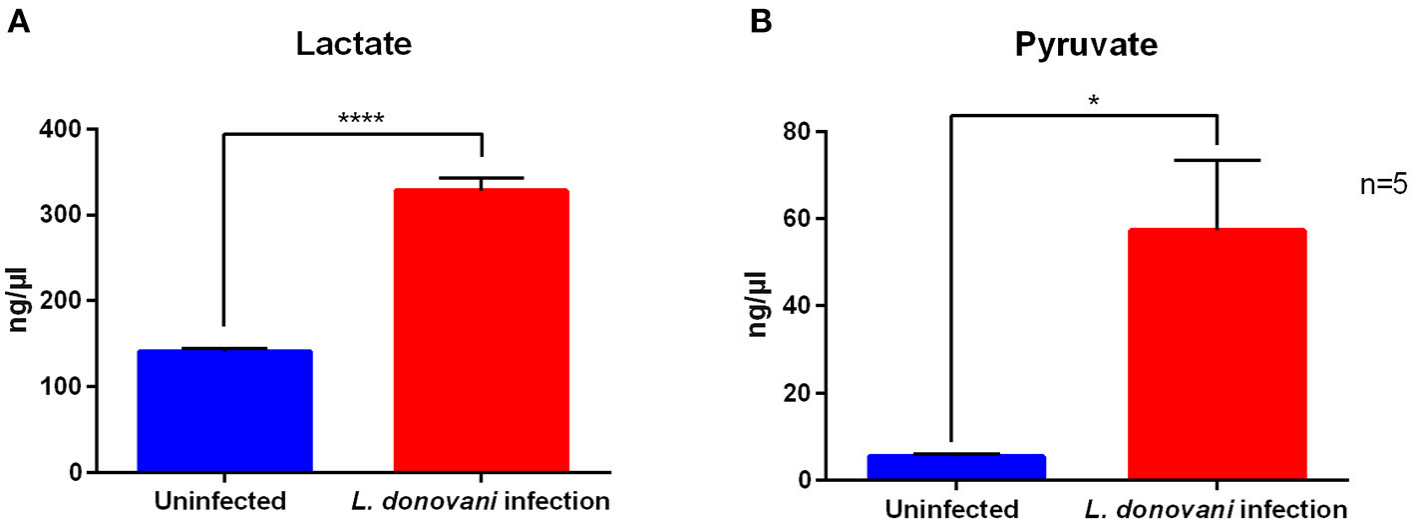
Lactate secretion and pyruvate content of *L. donovani*-infected neutrophils. Primary human neutrophils were infected with *L. donovani* promastigotes (ratio 1:10) for 3 h at 37°C and 5% CO_2_. Uninfected cells served as control. The infection rate was determined by Giemsa staining of cytocentrifuged samples. Subsequently, free, non-ingested parasites were removed by washing. Whole cell lysates were generated after 6 h post infection by boiling cells at 90°C for 5 min. Cell-free supernatants were collected after 6 h post infection. Lactate was detected in culture supernatants by using a lactate assay kit. Pyruvate was detected in whole cell lysates by using a pyruvate assay kit. Bar diagrams show mean concentration of lactate **(A)** after subtraction of the medium blanks and pyruvate **(B)** calculated by interpolation from standard curve ± SD (*n* = 5), ^*^p ≤ 0.05, ^****^p ≤ 0.0001.

As extracellular flux analysis indicated an increased glycolytic metabolism in *L. donovani*-infected neutrophils, the metabolic end products of glycolysis under aerobic settings, pyruvate, and under anaerobic conditions, lactate, were determined by a pyruvate assay kit and a lactate assay kit, respectively. As shown in Figures 5A,B, the lactate secretion as well as the pyruvate content of *L. donovani*-infected neutrophils was significantly increased compared to uninfected neutrophils. The accumulation of the glucose end products lactate and pyruvate of *L. donovani*-infected neutrophils highlight the important role of the glycolytic metabolism in neutrophils during *L. donovani* infection.

### The ATP Content of *L. donovani*-Infected Neutrophils Is Diminished Upon Glycolysis Inhibition

**Figure 6:**
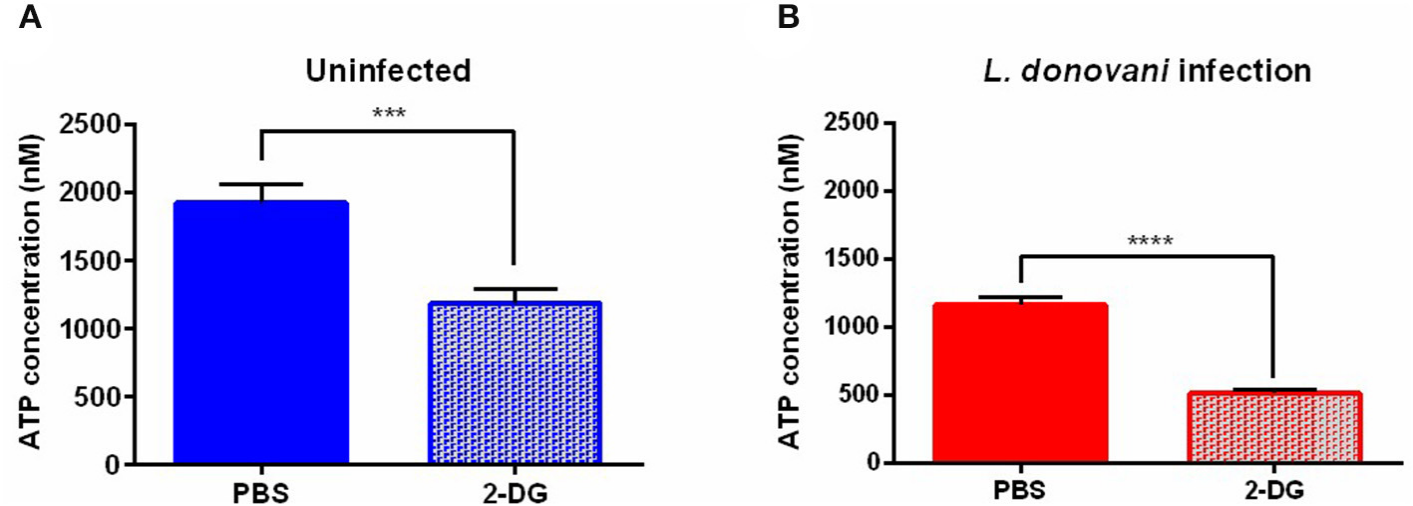
ATP concentration in *L. donovani*-infected neutrophils as response to metabolic inhibitors. Primary human neutrophils were infected with *L. donovani* promastigotes (ratio 1:10) for 3 h at 37°C and 5% CO_2_. Uninfected cells served as control. The infection rate was determined by Giemsa staining of cytocentrifuged samples. Subsequently, free, non-ingested parasites were removed by washing. After 6 h post infection cells were treated with 10 mM 2-DG for 3 h at 37°C and 5% CO_2_. PBS served as solvent control. Whole cell lysates were prepared and the ATP concentration was determined by using the ATP determination kit. Bar diagrams show the mean ATP concentration ± SD (*n* = 3) in uninfected **(A)** and *L. donovani*-infected neutrophils **(B)**, ^*^p ≤ 0.05, ^***^p ≤ 0.001, ^****^p ≤ 0.0001.

To confirm that glycolysis supplies neutrophils with energy during *L. donovani* infection, the ATP concentration in *L. donovani*-infected neutrophils was determined upon treatment with the glucose analog 2-deoxyglucose (2-DG), which leads to glycolysis impairment. After 6 hpi 2-DG treatment resulted in significantly decreased ATP concentrations of *L. donovani*-infected and uninfected neutrophils (Figures 6A–C). The observed decrease in the ATP concentration after 2-DG treatment complemented the previous results and highlighted the importance of glycolysis during *L. donovani* infection.

### Survival of *L. donovani* Promastigotes in 2-DG-Treated Neutrophils

**Figure 7:**
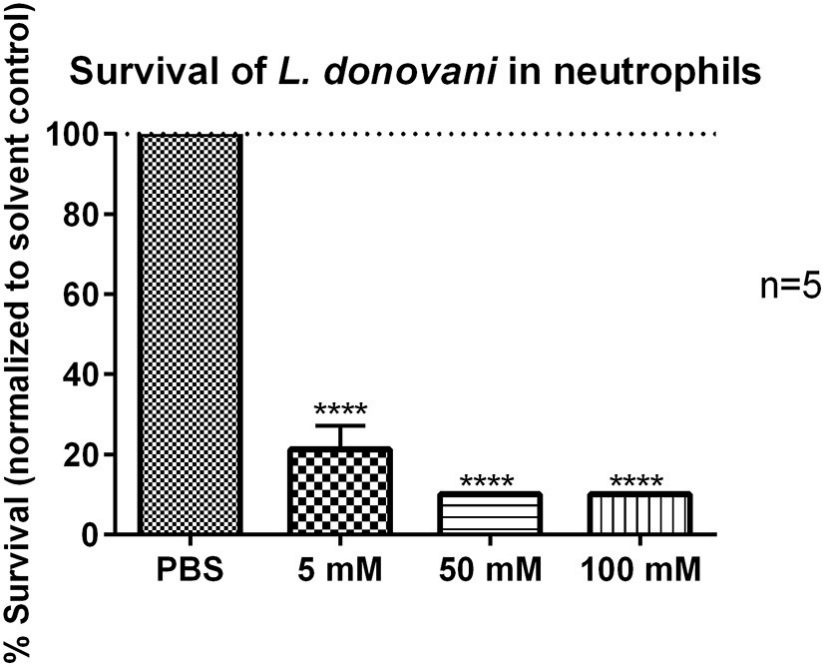
Survival of *L. donovani* promastigotes in 2-DG-treated neutrophils. Primary human neutrophils were infected with *L. donovani* promastigotes (ratio 1:10) for 3 h at 37°C, 5% CO_2_. The infection rate was determined by Giemsa staining of cytocentrifuged samples. After removing the free, non-ingested parasites the infected cells were treated with PBS, 5 mM, 50 mM, or 100 mM 2-DG. Survival of parasites was assessed after 24 h post infection by using the limiting dilution assay. The bar diagram shows the mean survival rates (%) normalized to PBS-treated control cells ± SD (*n* = 3), ^****^p ≤ 0.0001.

Considering the view that glycolytic metabolism plays an important role in neutrophils during *L. donovani* infection, inhibition of glycolysis with 2-DG was expected to abrogate antileishmanial effector functions in neutrophils. To further investigate this, *L. donovani*-infected neutrophils were treated with 5 mM, 50 mM, or 100 mM 2-DG (34–37) and the survival of *L. donovani* promastigotes was assessed by using the limiting dilution assay after 24 hpi. The non-toxic concentration range of 2-DG was previously determined by annexin-V PI staining (Supplementary Figure 1). As shown in Figure 7 the treatment of *L. donovani*-infected neutrophils with 5 mM or 50 mM 2-DG resulted in a significantly reduced survival rate of *L. donovani* promastigotes. These data indicated that the inhibition of glycolysis by 2-DG in *L. donovani*-infected neutrophils results in a strong reduction of parasite load, which is, however, unexpected.

### 2-DG Treatment Reduces ROS Production of *L. donovani*-Infected Neutrophils

**Figure 8:**
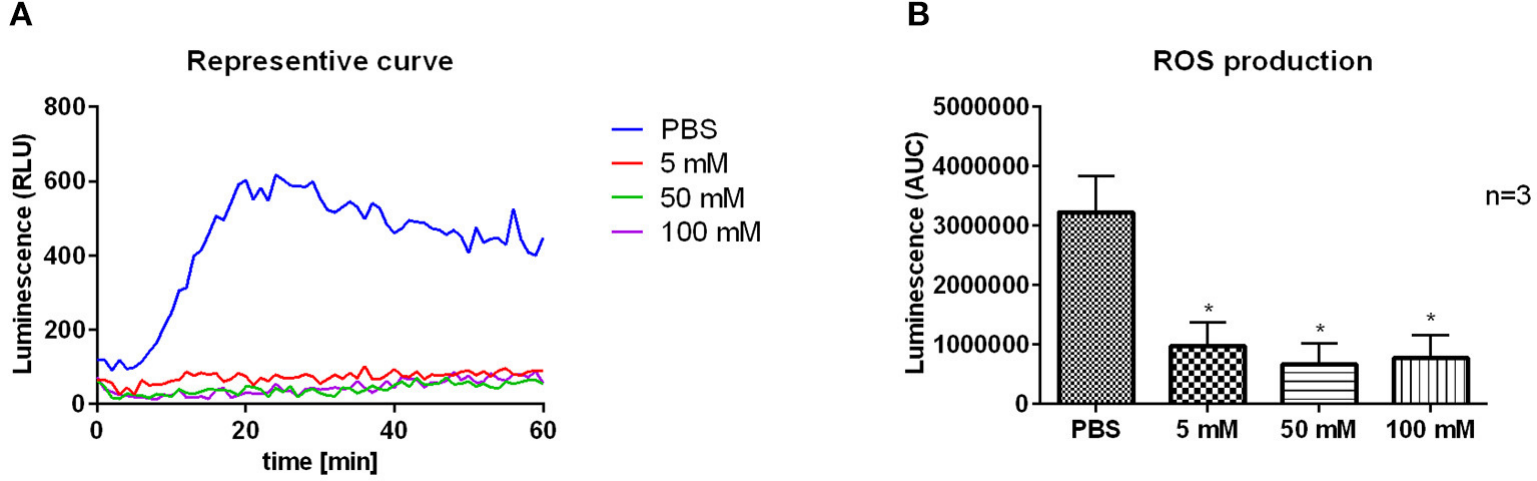
ROS production of 2-DG-treated *L. donovani*-infected neutrophils. Primary human neutrophils were infected with *L. donovani* promastigotes (ratio 1:10) for 3 h at 37°C, 5% CO_2_. The infection rate was determined by Giemsa staining of cytocentrifuged samples. After removing the free, non-ingested parasites the infected and uninfected cells were treated with PBS, 5 mM, 50 mM, or 100 mM 2-DG. After 24 h post infection the MPO-derived ROS production was measured for 1 h at 37°C and 5% CO_2_ after the stimulation with 20 nM PMA by using the luminol-based chemiluminescence assay. A representative curve of luminol chemiluminescence is shown in panel **(A)**. The bar diagram **(B)** shows the mean area under the curve (AUC) values ± SD (*n* = 3), ^*^p ≤ 0.05.

As an approach to clarify the unexpected finding, that 2-DG treatment reduced the parasitic load in neutrophils, we tested whether the decreased survival of *L. donovani* promastigotes in 2-DG-treated neutrophils is linked to enhanced ROS production. By using the luminol-based chemiluminescence assay of 2-DG treated *L. donovani*-infected neutrophils we observed that after treatment with 2-DG *L. donovani*-infected neutrophils showed significantly reduced ROS production compared to PBS-treated cells (Figures 8A,B). This data demonstrates that the inhibition of glycolysis by 2-DG in *L. donovani*-infected neutrophils does not increase the ROS production. Therefore, the reduced survival of the parasites in 2-DG treated neutrophils is not a result of enhanced ROS-mediated killing of the parasite.

### 2-DG Treatment Reduces the Survival of *L. donovani* Promastigotes

**Figure 9:**
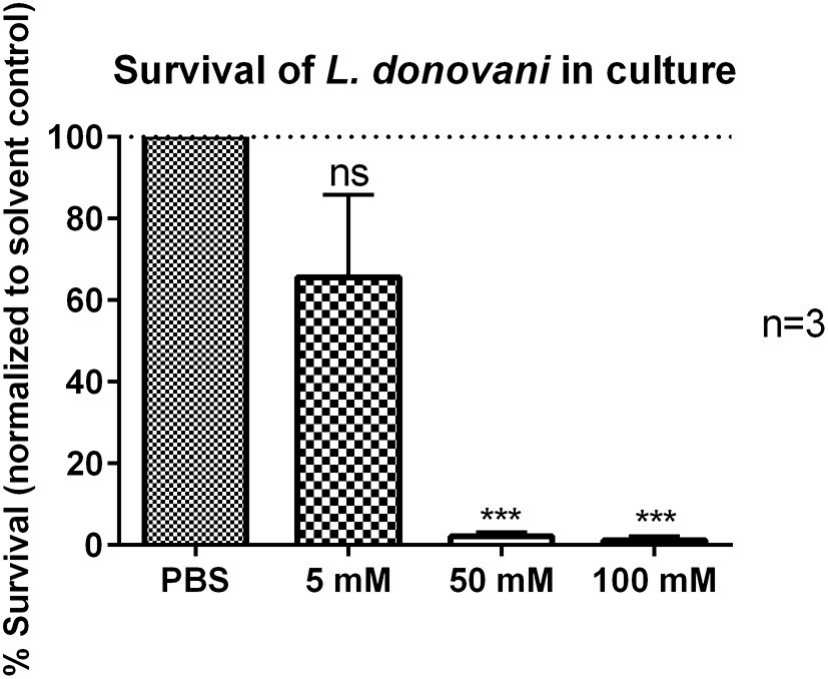
Survival of *L. donovani* promastigotes in the presence of 2-DG. *L. donovani* promastigotes were adjusted to 25 x 10^6^ cells/ml in complete medium and treated with 5 mM, 50 mM, or 100 mM 2-DG for 24 h at 37°C and 5% CO_2_. PBS treatment served as solvent control. Survival of parasites was assessed after 24 h by using the limiting dilution assay. The bar diagram shows the mean normalized to PBS-treated control cells ± SD (*n* = 3), ^***^p ≤ 0.001, ns, not-significant.

Next, it was tested if the reduced survival of *L. donovani* promastigotes in 2-DG-treated neutrophils was due to the direct cytotoxicity of 2-DG toward the parasite itself. *L. donovani* promastigotes were treated with 5 mM, 50 mM, or 100 mM 2-DG for 24 h and their survival was assessed by using the limiting dilution assay. As shown in Figure 9, treatment with 50 mM or 100 mM 2-DG resulted in a significantly reduced survival of *L. donovani* promastigotes. These data demonstrated that 2-DG is directly cytotoxic for *L. donovani* promastigotes.

### Impairment of Glycolytic Pathway *in vivo* Increases the Susceptibility to *L. donovani* Infection

**Figure 10:**
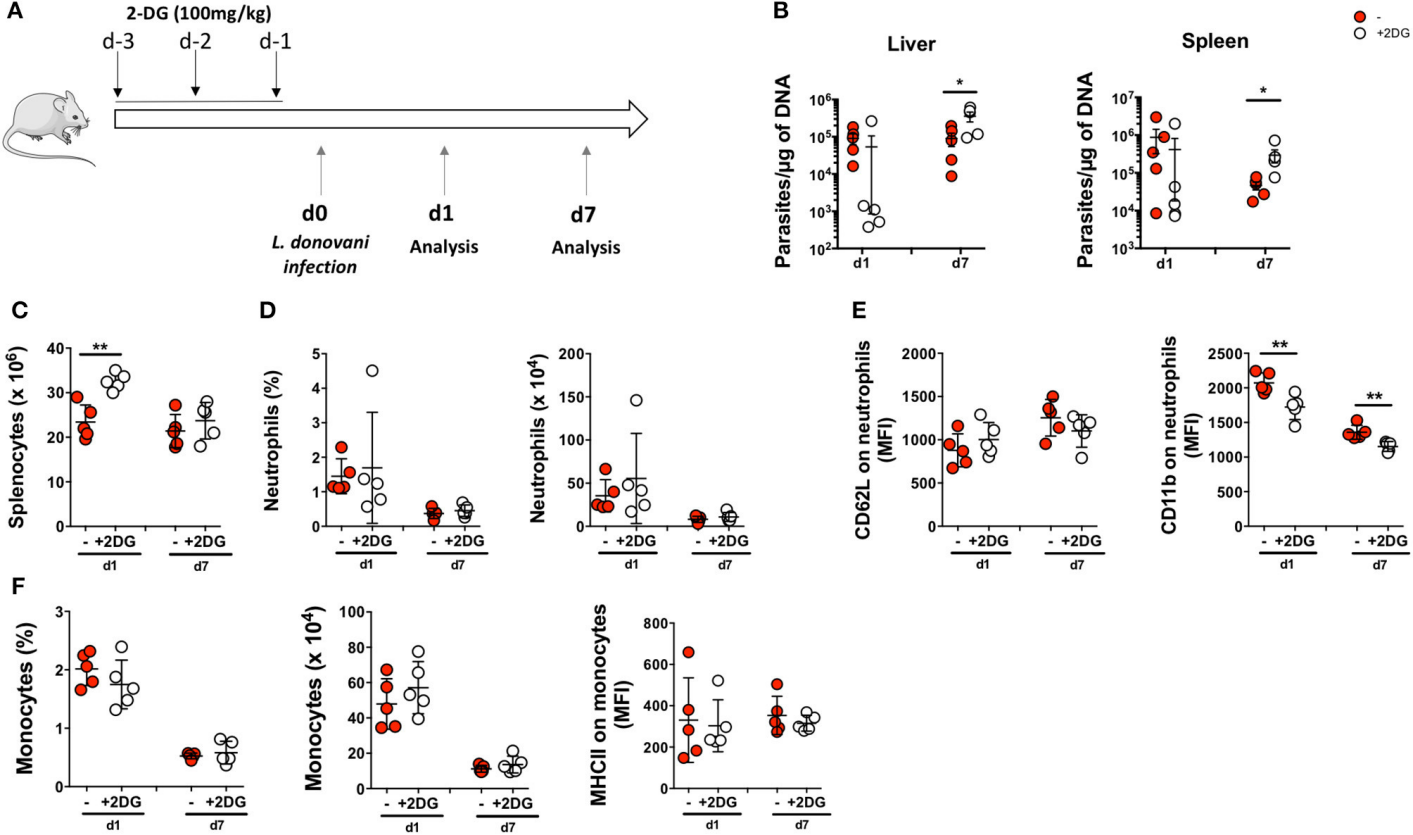
Impairment of glycolytic pathway *in vivo* increases susceptibility to *L. donovani* infection. **(A)** Mice were pre-treated with 2-deoxyglucose (2-DG) on days −3, −2 and −1 before infection. Untreated and 2-DG-treated mice were then infected intraperitoneally with 1 x 10^8^ *L. donovani* promastigotes and mice were euthanized at day 1 (d1) and day 7 (d7) post-infection. **(B)** Parasite burden in the liver and the spleen of infected animals at day 1 and day 7 post-infection. **(C)** The total number of splenocytes were accessed in untreated and 2-DG-treated mice. **(D)** The percentage and number of neutrophils were quantified in the spleen. **(E)** Expression of CD62L and CD11b were evaluated in neutrophils of infected animals at both timepoints. Data is expressed as mean ± SD. ^*^p < 0.05; ^**^p < 0.01; ^***^p < 0.001. **(F)** The percentage and number of monocytes were quantified in the spleen. Expression of MHCII was evaluated in monocytes of infected animals at both time points (d1 and d7). Data is expressed as mean ± SD. ^*^p < 0.05; ^**^p < 0.01.

As glucose metabolism is upregulated in *L. donovani*-infected neutrophils, we next investigated the impact of modulating glucose metabolism on *L. donovani* infection *in vivo*. Mice were pre-treated with the glucose analog 2-DG on day −3, −2, and −1 and of intraperitoneal infection with *L. donovani* promastigotes (Figure 10A). The impairment of glycolysis *in vivo* did not significantly affect the parasite load in liver and spleen at day one post-infection (Figure 10B). Yet, at day seven post-infection, we detected a significantly enhanced parasite load on the spleen and liver of 2-DG-treated mice, which demonstrates that glycolysis is crucial for host protective response (Figure 10B). Indeed, the median parasite burden on 2-DG-treated animals increased by 412- and 14-fold in the liver and spleen, respectively, from day 1 to 7. Although the number of splenocytes has increased in 2-DG-treated animals at day one post-infection when compared with the untreated animals, no major differences were observed in splenic numbers at day seven post-infection between the two groups (Figure 10C). Furthermore, 2-DG treatment did not result in major alterations in the numbers and percentage of neutrophils in spleen of infected animals (Figure 10D). We have also evaluated the expression of the activation and degranulation of CD62L and CD11b markers on splenic neutrophils. Although no major differences were observed in the expression of CD62L, a decrease in the surface expression of the degranulation marker was observed on neutrophils from 2-DG-treated animals at both time points when compared with the untreated counterparts (Figure 10E). Given that the surface expression of CD11b is associated with the activated state of neutrophils (38), the diminished CD11b expression is a sign for diminished activation or deactivation of neutrophils in 2-DG treated animals. The increased parasite burden in 2-DG-treated animals at day seven post-infection clearly correlates with the compromised anti-microbial functions of neutrophils.

Altogether, our results show that the inhibition of glycolysis *in vivo* is associated with increased *L. donovani* burden, an effect which correlates with reduced anti-parasitic activity of neutrophils.

## Discussion

The control of visceral leishmaniasis is challenged by the widespread emergence of resistance and treatment failure. A better understanding of the host-cell metabolism during *Leishmania* infection is essential to define potential targets for host-directed therapies. Our previous studies provided evidence that *Leishmania infantum*-infected macrophages showed an increased glycolytic profile during early infection (19). Neutrophils play either a protective or a detrimental role during the onset of leishmaniasis. Whether differences in cellular metabolism of neutrophils are associated with these differential functions is still unclear. Herein, our objective was to assess the metabolic profile of *Leishmania donovani*-infected neutrophils. The transcriptome profile of *L. donovani*-infected primary human neutrophils showed that genes for key enzymes of glycolysis are highly expressed and consistently and significantly upregulated in infected neutrophils 6 h after infection (Figure 1).

Based on the transcriptomic signature of *L. donovani*-infected neutrophils, we investigated the bioenergetics profile of *L. donovani*-infected neutrophils by using extracellular flux analysis. Six hours post-infection, higher basal extracellular acidification rate (ECAR) levels were observed in *L. donovani* infected neutrophils compared to uninfected neutrophils (Figure 2A). The key glycolytic parameters glycolysis and glycolytic capacity were significantly upregulated in *L. donovani*-infected neutrophils. At the same time point, higher basal oxygen consumption rate (OCR) values were observed for *L. donovani*-infected neutrophils compared to uninfected cells (Figure 3A). We found that without exception all respiratory parameters calculated from the OCR profile were upregulated in *L. donovani*-infected neutrophils (Figures 3B–E). However, no enhanced oxidative phosphorylation-derived ATP production was observed in *L. donovani*-infected neutrophils. Therefore, we can conclude that no metabolic switch from glycolysis to oxidative phosphorylation, as described for *L. infantum*-infected murine macrophages (19), occurs in *L. donovani*-infected neutrophils. Moreover, it is likely that the increased oxygen consumption of *L. donovani*-infected neutrophils is associated with enhanced ROS production as an effector mechanism against the intracellular pathogen. In line with our data, in previous studies neutrophils were found to contain only few mitochondria (31), having low levels of oxidative phosphorylation and mitochondrial enzymes including glutamate dehydrogenase and fumarase (39, 40). Additionally, our assumption that *L. donovani*-infected primary human neutrophils favor glycolysis for ATP production instead of oxidative phosphorylation was further supported by the observation that the competitive glycolysis inhibitor 2-deoxyglucose (2-DG) significantly reduced the ATP levels of *L. donovani*-infected primary human neutrophils (Figure 6).

Next, we investigated the glycolytic flux in *L. donovani*-infected primary human neutrophils in terms of glucose uptake and measurement of the glycolytic end products lactate and pyruvate. Both glucose uptake and accumulation of glycolytic end products were significantly upregulated in *L. donovani*-infected primary human neutrophils compared to uninfected cells (Figures 4, 5A,B). These results strengthen our observation of a primarily glycolytic metabolism in *L. donovani*-infected primary human neutrophils.

At this point, however we could not exclude that the increase in glycolysis was a consequence of metabolic reprogramming of the neutrophils by the parasite to change the host nutritional status to its favor. Therefore, we tested the antileishmanial capacity of neutrophils after the treatment with 2-DG. The 2-DG treatment significantly reduced the survival of *L. donovani* promastigotes in neutrophils (Figure 7). However, the reduced survival of *L. donovani* parasites was not a consequence of increased ROS production by neutrophils as 2-DG treatment abrogated ROS production in *L. donovani*-infected neutrophils (Figures 8A,B). As 2-DG competitively blocks the production of glucose-6-phosphate (G6P) both glycolysis and the pentose phosphate pathway (PPP) are inhibited in 2-DG-treated cells (37). The PPP utilizes G6P for the production of NADPH, the substrate of NOX-2, the multi-subunit enzymatic complex responsible for the oxidative burst in neutrophils (41, 42). Furthermore, Rice et al. (42) showed that the 2-DG mediated block of glycolysis reduces especially the initial-earlier phase oxygen consumption in neutrophils suggesting the requirement of glucose metabolism for the initiation of intense ROS production. Therefore, treatment with 2-DG certainly does not enhance ROS-production and consequent ROS-mediated killing of *Leishmania* in neutrophils. Still we observed a strongly reduced parasite load in neutrophils after 2-DG treatment. A possible explanation of this unexpected finding could be that 2-DG itself affect directly the viability of *Leishmania*. To investigate this possibility, we tested the survival of 2-DG treated *L. donovani* promastigotes in culture and observed a significant reduction in parasite survival (Figure 9). Therefore, we assume that an inhibition of glycolysis is toxic to the parasite in its promastigote form. Several studies showed that *Leishmania* promastigotes and amastigotes preferentially use glucose as carbon and energy source and even outsource glycolytic enzymes into specialized organelles, the glycosomes (44, 45).

Therefore, since both host neutrophils and *Leishmania* are highly dependent on glycolysis, an inhibition of glycolysis is not a promising approach as host-directed therapy. However, there was still the possibility to target glycolysis as antileishmanial therapy. The use of 2-DG in anti-cancer treatment is well accepted as tumor cells rely highly on aerobic glycolysis due to the Warburg effect (37, 38, 46). To test 2-DG as potential drug for anti-parasitic therapy, we pre-treated C57BL/6 mice with 2-DG and assessed the parasitic load in liver and spleen one day and seven days post infection. To our surprise, the parasitic load in liver and spleen was decreased, although not significant, at one day post infection but increased seven days post infection in 2-DG-treated animals (Figure 10B). The lower parasite burden in 2-DG-treated animals at day one post-infection is likely a consequence of the toxic effect of this compound on *Leishmania* parasites. The percentage and absolute number of neutrophils did not change upon 2-DG treatment (Figures 10C,D). However, the expression of the degranulation marker CD11b was decreased on neutrophils from 2-DG-treated animals (Figure 10E).

Since enhanced expression of CD11b is associated with the activated state of neutrophils (38), the diminished CD11b expression is a sign for diminished activation or deactivation of neutrophils in 2-DG treated animals. The increased parasite burden in 2-DG-treated animals at day seven post-infection, therefore, clearly correlates with the compromised anti-microbial functions of neutrophils. Since neutrophils are the first cells that encounter the parasite they are also the first cells affected by the 2-DG-mediated glycolysis inhibition in terms of antimicrobial response. As glycolysis drives the major effector functions in neutrophils, including phagocytosis (29, 30, 32), oxidative burst (31), degranulation (47), and the formation of NETs (48), it can be assumed that these effector functions are essential for the initial control of *L. donovani* infection. Consequently, inhibition of these glycolysis-driven effector functions results in excessive parasite proliferation. Although we did not detect any significant alterations on cell number or activation markers (Figure 10F), we cannot discard any potential 2-DG effect on other innate immune populations during the first week of infection, particularly the monocyte/macrophage population that may contribute to the parasite burden observed at day 7.

In summary, we demonstrate here that *L. donovani*-infected primary human neutrophils exert an increased glycolytic metabolism. Our study clearly shows that an inhibition of glycolysis results in impaired antileishmanial properties of neutrophils *in vitro* and enhanced parasite burden *in vivo*, highlighting the protective role of neutrophils in early *L. donovani* infection.

## Data Availability Statement

The raw transcriptome data has been uploaded and is available at EGA under data set IDs EGAD00001006807, EGAD00001006808 and EGAD00001006809 of study EGAS00001004912.

## Ethics Statement

The studies involving human participants were reviewed and approved by Ethical Committee of the University of Lübeck, Germany (18-186). The patients/participants provided their written informed consent to participate in this study. The animal study was reviewed and approved by University Minho (Portugal) Ethical Committee (process no. SECVS 074/2016) and complied with the guidelines of the Committee and National Council of Ethics for the Life Sciences (CNECV).

## Author Contributions

TL and RS designed the study. MO and CF conceived, designed and carried out the experiments. IW, AG, and HB performed the transcriptome analyses. MO, CF, RS, IW, HB, and TL wrote the manuscript. All authors read, edited and approved the final manuscript.

## Conflict of Interest

The authors declare that the research was conducted in the absence of any commercial or financial relationships that could be construed as a potential conflict of interest.

## Abbreviations

(D)PBS: (Dulbeccos')phosphate-buffered saline
2-DG: 2-deoxyglucose
2-NBDG: 2-deoxy-2-((7-nitro-2,1,3-benzoxadiazol-4-yl)amino)
AMPK: AMP-activated protein kinase
ANOVA: analysis of variance
ATP: adenosine triphosphate
CD11b: integrin alpha M
CD62L: L-selectin
CO_2_: carbon dioxide
ECAR: extracellular acidification rate
EDTA: ethylenediaminetetraacetic acid
FACS: fluorescence activated cells sorting
FCCP: carbonyl cyanide-4-(trifluoromethoxy)phenylhydrazone
FCS: fetal calf serum
G6P: glucose-6-phosphate
GLUT: glucose transporters
HEPES: (4-(2-hydroxyethyl)-1-piperazineethanesulfonic acid)
KHCO_3_: potassium bicarbonate
MPO: myeloperoxidase
NE: neutrophil elastase
NETs: neutrophil extracellular traps
OCR: oxygen consumption rate
PE: phycoerythrin
PMA: phorbol 12-myristate 13-acetate
PPP: pentose phosphate pathway
ROS: reactive oxygen species
RT: room temperature
SGLTs: sodium coupled glucose transporters
SIRT1: sirtuins 1
VL: visceral leishmaniasis.
