# Supplementary material for: Enhanced Glycolysis Is Required for Antileishmanial Functions of Neutrophils Upon Infection With Leishmania donovani
Source: Front Immunol. 2021 Mar 19;12:632512. doi: 10.3389/fimmu.2021.632512 (PMC8017142; doi:10.3389/fimmu.2021.632512)
Supplement: Supplementary file 1 [file Data_Sheet_1.PDF]

Supplementary Materials

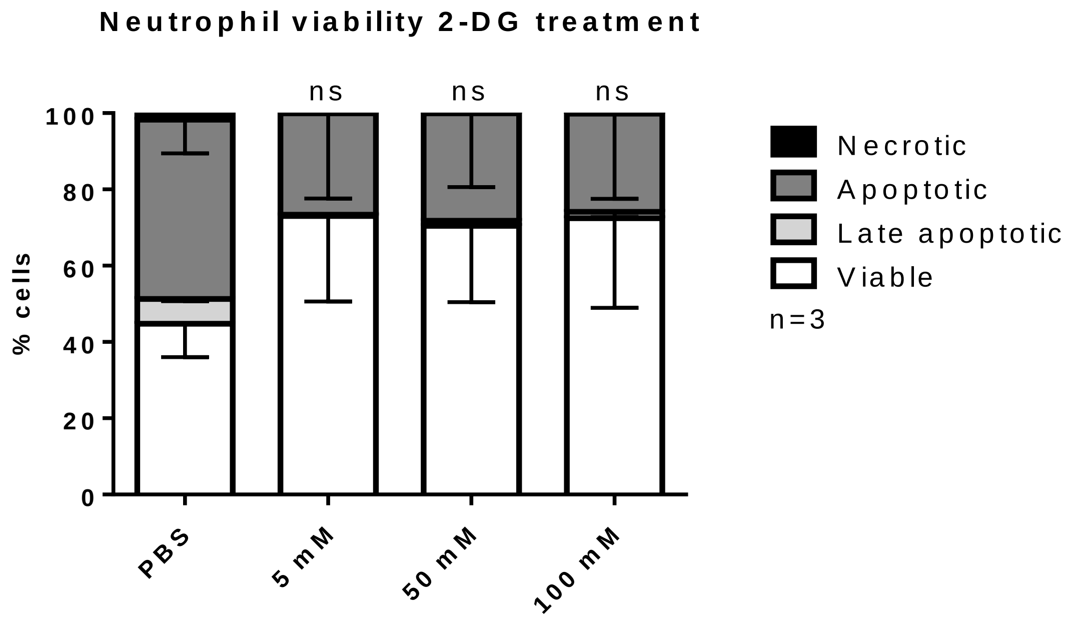

Supplementary Figure 1. Primary human neutrophils were treated with 5 mM, 50 mM or 100 mM 2-DG. PBS treatment served as solvent control. After 24 h apoptosis and cell viability were assessed by annexin-V and PI staining and analyzed by flow cytometry. The ratio (%) of viable cells was used for statistical analysis by one-way ANOVA. The bar diagram shows the mean ± SD (n=3), ns = not-significant.

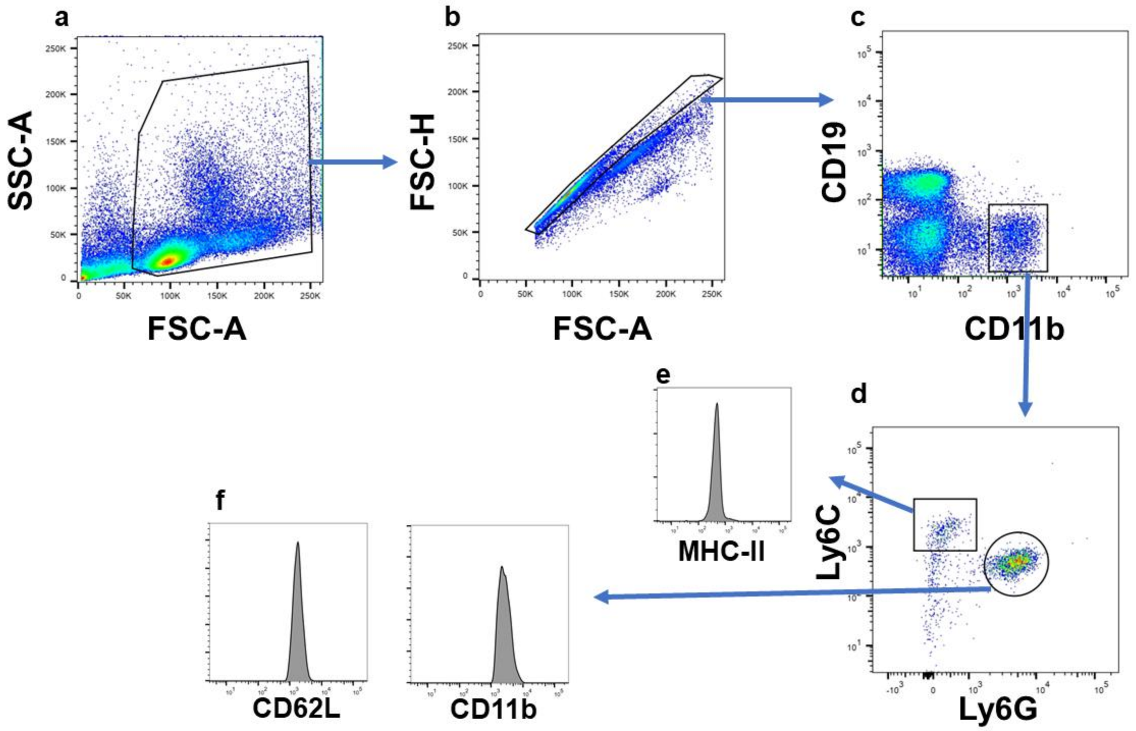

Supplementary Figure 2. Gating strategy for the definition of neutrophils and monocytes by using flow cytometry. The gating strategy is represented: (**a**) dead cells, debris and platelets were excluded in the first gating step. Subsequently, (**b**) singlets were selected for posterior analysis. Then, (**c**) myeloid cells were gated on CD11b positive cells. Of note, this gate excluded B cells (CD19 positive cells), T lymphocytes and other minor non-myeloid populations (CD19 and CD11b negative cells). (**d**) The monocyte and neutrophil populations were then gated on Ly6C and Ly6G positive cells. The mean fluorescent intensity of the surface expression of (**e**) MHC-II (on monocytes) and (**f**) CD62L and CD11b (on neutrophils) was finally quantified.

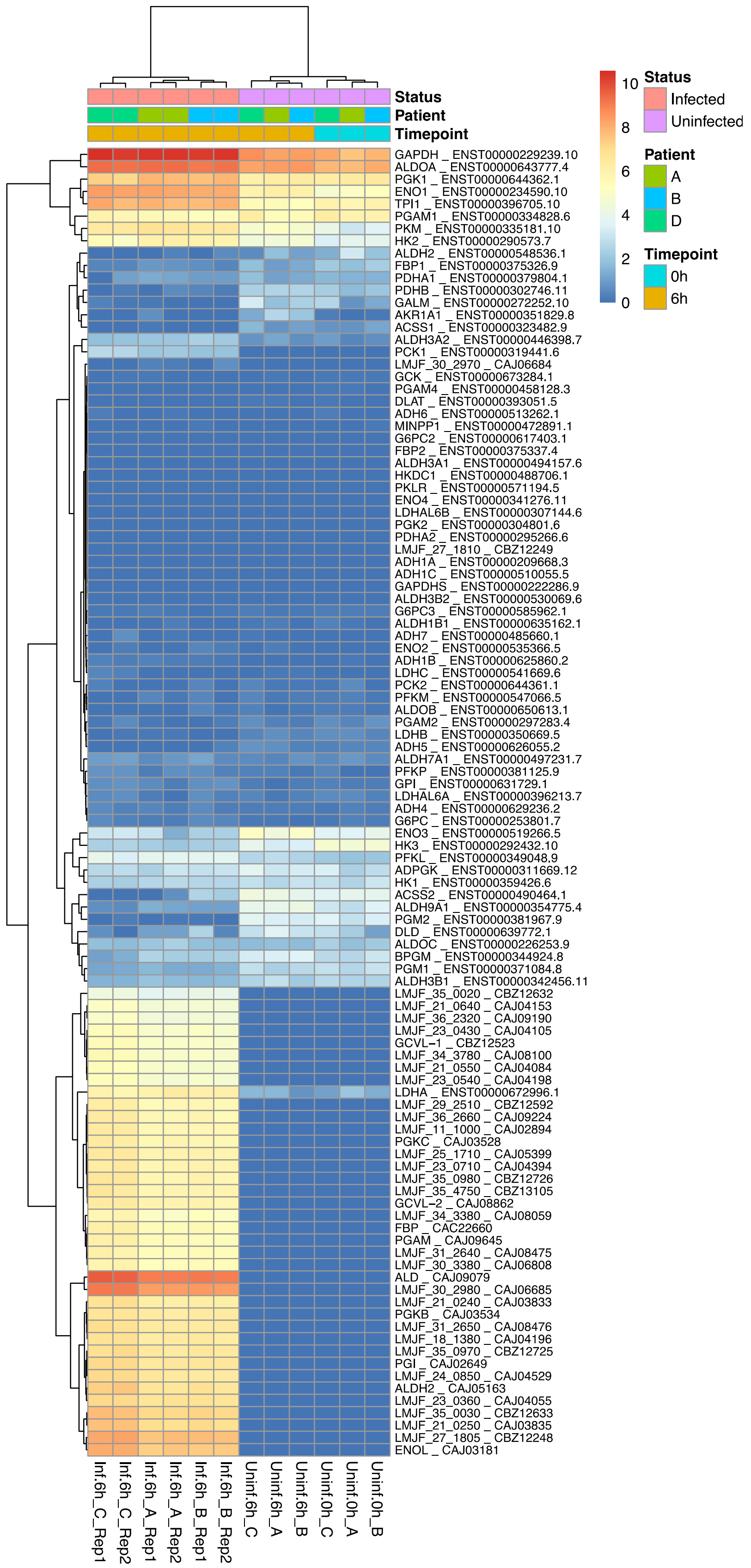
Status
Patient
Timepoint
10
8
6
4
2
0
Status
Infected
Uninfected
Patient
A
B
D
Timepoint
0h
6h
GAPDH _ ENST00000229239.10
ALDOA _ ENST00000643777.4
PGK1 _ ENST00000644362.1
ENO1 _ ENST00000234590.10
TPI1 _ ENST00000396705.10
PGAM1 _ ENST00000334828.6
PKM _ ENST00000335181.10
HK2 _ ENST00000290573.7
ALDH2 _ ENST00000548536.1
FBP1 _ ENST00000375326.9
PDHA1 _ ENST00000379804.1
PDHB _ ENST00000302746.11
GALM _ ENST00000272252.10
AKR1A1 _ ENST00000351829.8
ACSS1 _ ENST00000323482.9
ALDH3A2 _ ENST00000446398.7
PCK1 _ ENST00000319441.6
LMJF_30_2970 _ CAJ06684
GCK _ ENST00000673284.1
PGAM4 _ ENST00000458128.3
DLAT _ ENST00000393051.5
ADH6 _ ENST00000513262.1
MINPP1 _ ENST00000472891.1
G6PC2 _ ENST00000617403.1
FBP2 _ ENST00000375337.4
ALDH3A1 _ ENST00000494157.6
HKDC1 _ ENST00000488706.1
PKLR _ ENST00000571194.5
ENO4 _ ENST00000341276.11
LDHAL6B _ ENST00000307144.6
PGK2 _ ENST00000304801.6
PDHA2 _ ENST00000295266.6
LMJF_27_1810 _ CBZ12249
ADH1A _ ENST00000209668.3
ADH1C _ ENST00000510055.5
GAPDHS _ ENST00000222286.9
ALDH3B2 _ ENST00000530069.6
G6PC3 _ ENST00000585962.1
ALDH1B1 _ ENST00000635162.1
ADH7 _ ENST00000485660.1
ENO2 _ ENST00000535366.5
ADH1B _ ENST00000625860.2
LDHC _ ENST00000541669.6
PCK2 _ ENST00000644361.1
PFKM _ ENST00000547066.5
ALDOB _ ENST00000650613.1
PGAM2 _ ENST00000297283.4
LDHB _ ENST00000350669.5
ADH5 _ ENST00000626055.2
ALDH7A1 _ ENST00000497231.7
PFKP _ ENST00000381125.9
GPI _ ENST00000631729.1
LDHAL6A _ ENST00000396213.7
ADH4 _ ENST00000629236.2
G6PC _ ENST00000253801.7
ENO3 _ ENST00000519266.5
HK3 _ ENST00000292432.10
PFKL _ ENST00000349048.9
ADPGK _ ENST00000311669.12
HK1 _ ENST00000359426.6
ACSS2 _ ENST00000490464.1
ALDH9A1 _ ENST00000354775.4
PGM2 _ ENST00000381967.9
DLD _ ENST00000639772.1
ALDOC _ ENST00000226253.9
BPGM _ ENST00000344924.8
PGM1 _ ENST00000371084.8
ALDH3B1 _ ENST00000342456.11
LMJF_35_0020 _ CBZ12632
LMJF_21_0640 _ CAJ04153
LMJF_36_2320 _ CAJ09190
LMJF_23_0430 _ CAJ04105
GCVL−1 _ CBZ12523
LMJF_34_3780 _ CAJ08100
LMJF_21_0550 _ CAJ04084
LMJF_23_0540 _ CAJ04198
LDHA _ ENST00000672996.1
LMJF_29_2510 _ CBZ12592
LMJF_36_2660 _ CAJ09224
LMJF_11_1000 _ CAJ02894
PGKC _ CAJ03528
LMJF_25_1710 _ CAJ05399
LMJF_23_0710 _ CAJ04394
LMJF_35_0980 _ CBZ12726
LMJF_35_4750 _ CBZ13105
GCVL−2 _ CAJ08862
LMJF_34_3380 _ CAJ08059
FBP _ CAC22660
PGAM _ CAJ09645
LMJF_31_2640 _ CAJ08475
LMJF_30_3380 _ CAJ06808
ALD _ CAJ09079
LMJF_30_2980 _ CAJ06685
LMJF_21_0240 _ CAJ03833
PGKB _ CAJ03534
LMJF_31_2650 _ CAJ08476
LMJF_18_1380 _ CAJ04196
LMJF_35_0970 _ CBZ12725
PGI _ CAJ02649
LMJF_24_0850 _ CAJ04529
ALDH2 _ CAJ05163
LMJF_23_0360 _ CAJ04055
LMJF_35_0030 _ CBZ12633
LMJF_21_0250 _ CAJ03835
LMJF_27_1805 _ CBZ12248
ENOL _ CAJ03181
Inf.6h_C_Rep1
Inf.6h_C_Rep2
Inf.6h_A_Rep1
Inf.6h_A_Rep2
Inf.6h_B_Rep1
Inf.6h_B_Rep2
Uninf.6h_C
Uninf.6h_A
Uninf.6h_B
Uninf.0h_C
Uninf.0h_A
Uninf.0h_B

Supplementary Figure 3. Heatmap of all glycolytic genes, i.e. genes mapping to the KEGG pathway glycolysis for six samples without and six samples 6 hours after *L. donovani* infection. Displayed expression values have been normalized across samples and are provided as log2 transcripts per million (TPM). Transcripts starting with ENST are human, others refer to *L. major*. Of every gene, only the transcript with maximum mean normalized TPM value is shown.
